# Determining the stability of minimally displaced lateral humeral condyle fractures in children: ultrasound is better than arthrography

**DOI:** 10.1186/s13018-020-02174-8

**Published:** 2021-01-09

**Authors:** Xing Wu, Xiongtao Li, Shaowei Yang, Si Wang, Jingdong Xia, Xiaoliang Chen, Xiantao Shen

**Affiliations:** 1 Department of Pediatric Orthopedic Surgery, Wuhan Children’s Hospital (Wuhan Maternal and Child Healthcare Hospital), Tongji Medical College, Huazhong University of Science & Technology, 100 Hong-Kong road, Wuhan, 430016 People’s Republic of China; 2 Department of Radiology, Wuhan Children’s Hospital (Wuhan Maternal and Child Healthcare Hospital), Tongji Medical College, Huazhong University of Science & Technology, 100 Hong-Kong road, Wuhan, 430016 China

**Keywords:** Ultrasound, Arthrography, Lateral condyle fractures, Children

## Abstract

**Background:**

Evaluating of the articular cartilage status of the distal humeral epiphysis is difficult. Ultrasound imaging of the elbow is increasingly being used to confirm the integrity of the articular cartilage of minimally displaced lateral humeral condyle fractures in children with minimally displaced fractures. The aims of this study were to assess the correlations between ultrasound and arthrography findings for predicting the integrity of the cartilage hinge and to describe the utility of ultrasound in determining the need for pre-treatment.

**Methods:**

Thirty-nine patients with minimally displaced lateral humeral condyle fractures who underwent ultrasound and arthrography examinations before surgery from May 2018 to December 2019 were included in this study. Ultrasound and arthrography predictors of the cartilage hinge status were independently measured. The ultrasound and arthrography results were compared.

**Results:**

The mean displacement of the fractures was 3.1 mm (range, 2.0~5.0 mm). Arthrography showed incomplete fractures in 24 patients (61.5%) and complete fractures in 15 patients (38.5%). Ultrasound showed incomplete fractures in 25 patients (64.1%) and complete fractures in 14 patients (35.9%). The ultrasound and arthrography results of the integrity of the articular surface were consistent in 92.3% of the cases, including 23 that were predicted to have an intact articular surface and 13 that were predicted to have an incongruity articular surface. There was no correlation between the displacement and the fracture appearing complete on the ultrasound scan. The Pearson coefficient between ultrasound and arthrography for assessing the integrity of the articular surface was 0.837.

**Conclusions:**

Ultrasound and arthrography assessments of the integrity of the cartilage hinge status appear to be highly consistent. Ultrasound can be used as a complementary tool with arthrography to predict the integrity of the cartilage hinge status in children with minimally displaced lateral humeral condyle fractures.

**Level of evidence:**

Prospective study; level II.

## Background

Lateral humeral condyle fractures (LHCFs) are the second most common elbow fractures in children, accounting for 12–20% of elbow fractures [1]. According to published guidelines, the indication for treatment is dependent on the displacement and stability of the fracture. It is still controversial whether it is necessary to perform open reduction and internal fixation (ORIF) for minimally displaced LHCFs with an incongruent articular surface. ORIF is associated with complications, including avascular necrosis, premature physeal closure, non-union, arthrofibrosis, infection, wound scars, and refracture [2, 3]. Closed reduction and percutaneous pinning (CRPP) is a safe and effective alternative treatment for minimally displaced LHCFs with an intact articular surface [3, 4].

The integrity of the articular cartilage of the distal humeral epiphysis determines the stability of the LHCF [5]. If the cartilage of the hinge is not intact, the fracture is complete, and the injury is unstable and predisposed to further displacement. Standard radiography has limitations in showing the epiphyseal cartilage of the distal humerus in children. Magnetic resonance imaging (MRI) [6, 7] or arthroscopy [8, 9] can assess the integrity of the articular cartilage, but the former method requires sedation. It is difficult for children to cooperate during the examination, so arthroscopy requires general anesthesia. Arthrography is mainly used to assess the integrity of the articular cartilage in these fractures. However, arthrography also requires sedation and may lead to invasive infections. Therefore, it is exceedingly essential to evaluate the articular cartilage status before treatment. Recently, ultrasound has been shown to be valuable in assessing the stability of fractures [10–12]. However, no data exist on the correlation between arthrography and ultrasound findings in assessing the integrity of articular cartilage with minimally displaced fractures.

The purposes of this study were to assess the correlation between ultrasound and arthrography findings for predicting the integrity of the cartilage hinge and to describe the utility of ultrasound in determining the need for pre-treatment for minimally displaced LHCFs in children.

## Methods

The institutional review board approved this prospective study, and the patients and their parents gave informed consent. Patients diagnosed with a minimally displaced LHCF (2~5 mm) between May 2018 and December 2019 participated in the study. Patients with an open injury or multiple injuries, fractures with a displacement of more than 5 mm, or an elbow with obvious soft tissue swelling and patients older than 9 years old with trochlear epiphyseal ossification were excluded. All patients underwent reduction and fixation under general anesthesia. Ultrasound and arthrography were performed before and after reduction. The results of the ultrasound and arthrography analyses were considered separately, and the experts were blinded to each other’s results.

**Fig. 1:**
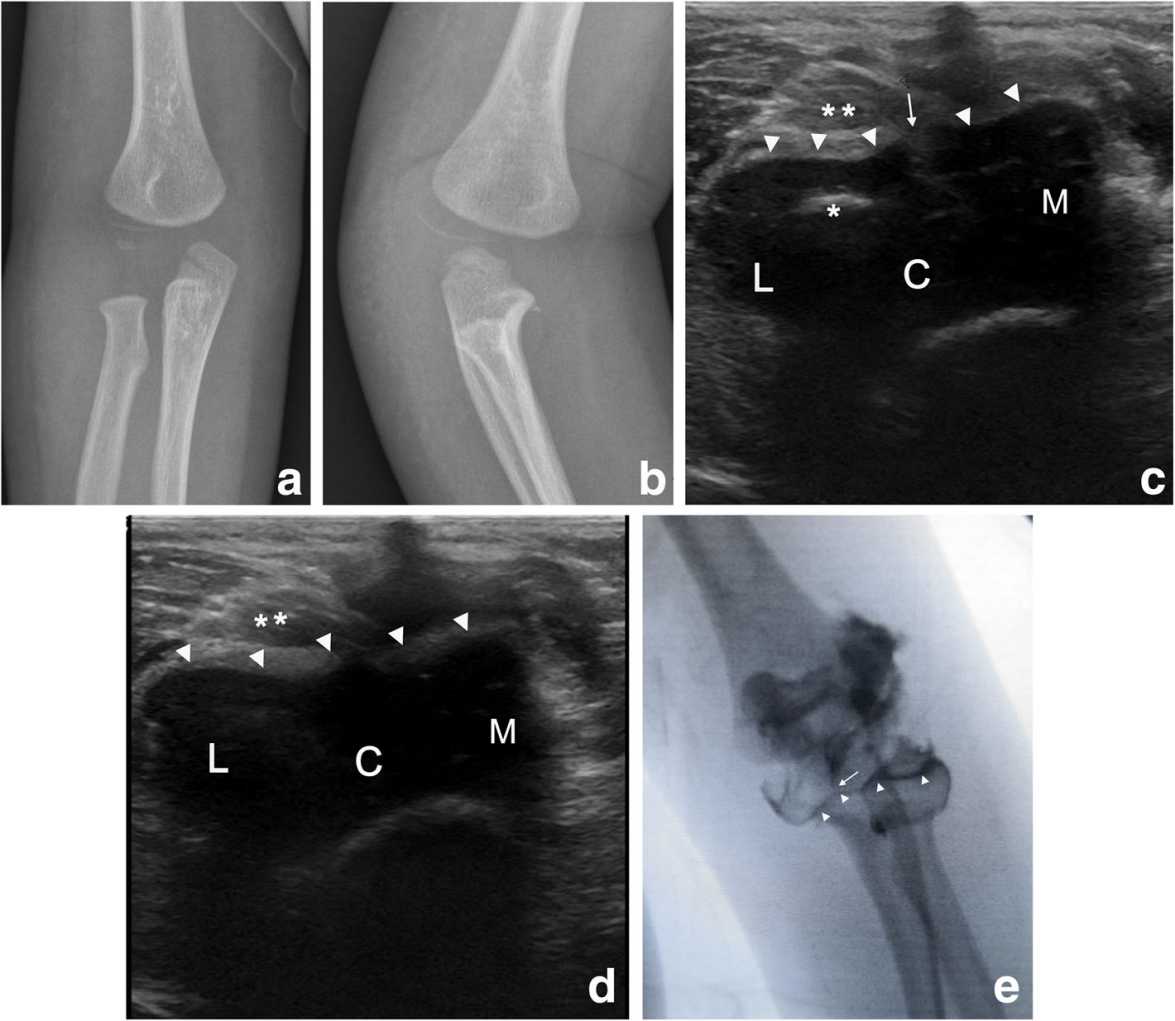
Patient case 24. **a** The anteroposterior radiographs showed a displacement of 2.5 mm. **b** The internal oblique radiographs showed a displacement of 5 mm. **c** The transverse ultrasound image showed the disrupted cartilage hinge on lays when the center of the ossification region in the capitellum epiphysis was found. **d** The transverse ultrasound image showed the intact cartilage hinge before reaching the articular surface and the disrupted cartilage hinge (arrow). **e** Radiographs in the anteroposterior views of an arthrogram. The dye was tracked along the fracture line and stopped before reaching the articular surface (arrow), thus indicating an intact cartilaginous hinge. Arrowhead, cartilage hinge; asterisk, epiphyseal core of ossification of the capitellum; double asterisks, hemorrhage; M, medial; L, lateral; C, cartilage of the epiphysis

**Fig. 2:**
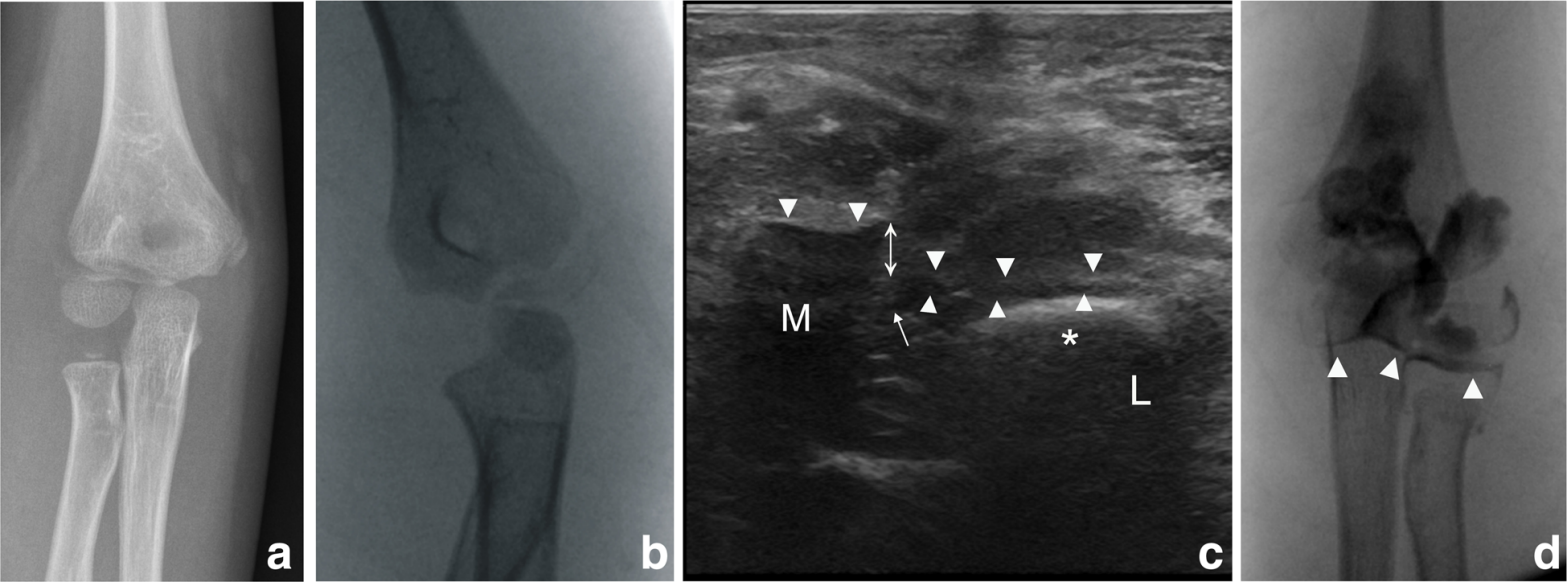
Patient case 4. **a**, **b** Radiographs in the anteroposterior and internal oblique views. **c** The transverse ultrasound image showed the disrupted cartilage hinge and the stair sign in a large cartilage gap. **d** Radiographs of anteroposterior views of an arthrogram. The dye continued along the fracture line and passes through the articular surface, thus indicating an incongruity of the articular surface. Arrow, disrupted cartilage hinge; arrowhead, cartilage hinge; Asterisk, epiphyseal core of ossification of the capitellum; M, medial; L, lateral

The study was designed so that all patients underwent ultrasound first with a GE LOGIQ e ultrasound system (GE Healthcare, Milwaukee, WI, USA) equipped with 7.0–12.5 MHz linear array transducer (GE Healthcare, Tokyo). The ultrasound examinations and analyses were conducted by one pediatric orthopedic surgeon with experience in osteosonographic diagnostics in children. Ultrasonographic imaging of the distal humerus was performed and documented in five standardized sectional planes: (1) the ventral-radial, (2) ventral-median, (3) dorsal-radial, (4) lateral, and (5) anterior transversal planes [10, 11, 13]. With the patient’s elbow placed in extension, the transducer was placed on the anterior aspect of the distal humerus to assess the transverse section. The ultrasound transducer was slid around the center of the ossification region of the capitellum epiphysis, and the cartilage hinge was located between the ossification region of the capitellum and the far end of the articular cartilage. The integrity of the articular cartilage at the distal humeral epiphysis was determined in the anterior transversal view. As we described previously [11], an intact articular surface was defined as that in which the fracture line was limited to the articular cartilage, and the articular cartilage of the distal humerus was continuous (Fig. 1). An incongruent articular surface was defined as that in which the fracture line extended through the cartilaginous epiphysis into the elbow joint, the articular cartilage was displaced, and the hyperechoic gap could be seen (Fig. 2).

An arthrography scan was performed after the ultrasound scan. Arthrography was performed by inserting the contrast dye into the elbow joint [14]. A 22-gauge needle was inserted directly posterior into the olecranon fossa when the elbow was flexed to 90°. A volume of 0.5–2 ml of iohexol contrast was injected into the joint, and the joint was moved through a range of motion. Fluoroscopic images were obtained to locate the articular cartilage of the distal humerus. The images were analyzed by an experienced pediatric orthopedic surgeon (S.T.), who was blinded to the ultrasound results. The integrity of the cartilage hinge of the distal humerus was independently measured on the radiographs and ultrasound images by two observers. The repeated assessments of the elbow fracture yielded an interrater correlation coefficient higher than 0.8.

After the fracture was confirmed to be reduced to within 2 mm and the articular cartilage of the distal humeral was continuous, CRPP was performed. If the fracture reduction procedure did not reduce the displacement to within 2 mm or the articular cartilage of the distal humeral was displaced, ORIF was performed. A long-arm cast was used in all patients, and the pins were removed 4 to 6 weeks after surgery.

### Statistical analysis

For the descriptive analysis, percentages were calculated for the categorical variables, and averages were calculated for the quantitative variables. The Spearman correlation coefficient was used to analyze the relationship between fracture displacement and the integrity of the articular surface and the relationship between ultrasound and arthrography measurements. *P* < 0.05 was considered statistically significant. The statistical analysis was performed with the SPSS software, version 19.0 (IBM Corporation, Armonk, NY).

## Results

A total of 39 patients with an average age of 53.9 months (range, 18~102 months) were included in the study. There were 27 boys and 12 girls. There were 22 left fractures and 17 right fractures. According to the initial radiographs, the mean displacement of the fractures was 3.1 mm (range, 2.0~5.0 mm). The results are shown in Table 1.

**Table 1:** Patient demographic data

ID	Sex	Age (month)	Side	Radiographic displacement (mm)	Time to examination (d)	Integrity of cartilage hinge arthrography	Integrity of cartilage hinge US
1	Female	32	Right	3.56	3	I	I
2	Male	52	Right	3.42	1	I	I
3	Male	52	Left	2.4	1	I	D
4	Female	72	Right	3.7	3	D	D
5	Male	75	Left	3.9	3	D	I
6	Male	49	Right	2.2	3	I	I
7	Male	80	Right	4	4	D	D
8	Male	85	Left	3.99	3	D	D
9	Male	30	Right	2.4	4	I	I
10	Male	24	Right	3.96	3	I	I
11	Female	21	Left	3.51	2	I	I
12	Male	42	Right	2	3	I	I
13	Male	38	Right	3.3	3	I	I
14	Male	36	Left	2.6	3	D	D
15	Male	93	Left	3.2	2	I	I
16	Male	56	Left	3.3	1	D	D
17	Male	55	Right	2.4	3	I	I
18	Male	64	Left	2.1	4	I	I
19	Male	92	Left	2.6	3	I	I
20	Female	85	Left	3.4	3	D	D
21	Female	49	Left	2.1	2	D	D
22	Female	18	Left	2.3	3	I	I
23	Female	24	Right	2.9	1	I	I
24	Male	18	Right	5	3	I	I
25	Male	38	Right	2.3	2	I	I
26	Male	36	Left	3.8	2	D	D
27	Male	93	Right	2.5	3	I	I
28	Male	55	Left	2.4	4	I	I
29	Female	85	Left	3.3	2	D	D
30	Male	102	Left	3	3	D	D
31	Female	18	Left	2.5	2	I	I
32	Female	24	Right	3.1	3	I	I
33	Male	54	Left	2.6	1	I	I
34	Female	32	Left	3.7	3	D	D
35	Male	90	Right	3.4	3	D	I
36	Male	82	Left	2.3	3	I	I
37	Male	51	Left	2.4	3	I	I
38	Male	43	Right	3.5	2	D	D
39	Female	55	Left	4.2	4	D	D

*I* intactness, *D* disruption

The operation was performed an average of 2.7 days (range, 1~5 days) after the fracture. Arthrography analysis was performed for all patients. The fracture was incomplete in 24 patients (61.5%) and complete in 15 patients (38.5%). There was a correlation between the displacement and fracture being complete (*P* < 0.05). The ultrasound examination took an average of 3 min. The fracture was incomplete in 25 patients (64.1%) and complete in 14 patients (35.9%). There was no correlation between displacement and the fracture being complete (*P* > 0.05).

Ultrasound and arthrography predicted the same outcomes regarding the integrity of the articular surface in 36 (92.3%) of the 39 patients; among these 36 patients, 23 were predicted to have intact articular surfaces, and 13 were predicted to have incongruity articular surfaces. The correlation coefficient between these imaging modalities was 0.837 (*P* < 0.05). Of the three patients with > 4 mm of fracture displacement, one was found to have an incomplete articular surface, and two were found to have a complete articular surface.

Among the three patients with different prediction results, patient number 3 had a displacement of 2.4 mm and was predicted to have an intact articular surface by ultrasound but an incongruous incongruity articular surface by arthrography. Patient number 5 had a displacement of 3.9 mm and was predicted to have an incongruous articular surface by ultrasound but an intact articular surface by arthrography. Patient number 35 had a displacement of 3.4 mm and was predicted to have a congruous articular surface by ultrasound but an incongruous articular surface by arthrography.

All cases were treated with CRPP, the average magnitude of postoperative displacement was less than 2 mm, and the articular cartilage of the distal humerus was continuous on ultrasound. Fracture union was achieved, at which point the cast was removed. There were no cases of non-union.

## Discussion

LHCF is an intra-articular fracture. The integrity of the articular cartilage is an essential factor in predicting the stability of a fracture. In the present study, both ultrasound and arthrography were used to assess the preoperative integrity of the articular cartilage status, and the results were highly consistent. Compared with arthrography, ultrasound was more efficient in determining the integrity of the articular cartilage noninvasively, without ionizing radiation, and ultrasound was more convenient to use. Ultrasound can be used as a complementary tool with arthrography to predict the integrity of the articular cartilage status in patients with minimally displaced LHCFs.

The classification systems recently proposed by Song et al. [15] and Weiss et al. [2] are based on the integrity of the articular cartilage surface. Because the distal humeral epiphysis is not ossified, the cartilage of the distal humerus cannot be detected by radiography. Therefore, there is controversy regarding the relationship between the integrity of the articular cartilage status and the results determined by the radiography. Ultrasound is a reliable, ionizing radiation-free, low-cost, noninvasive technique that does not require sedation or general anesthesia, especially for pediatric elbow examinations [10, 11, 13, 16]. A previous study showed that transverse ultrasound could be used to detect whether a fracture was complete or incomplete [11]. Vocke-Hell and Schmid [10] found that ultrasound can show whether the fracture line extends through the articular cartilage in the transversal view. If the hypoechoic cartilage hinge is disrupted and the hyperechoic fracture line extends to the distal humeral articular cartilage, the fracture is determined to be a complete LHCF. If the hypoechoic articular cartilage hinge is smooth and continuous, it is judged to be an incomplete LHCF. The present study showed that the ability of arthrography to predict the integrity of articular surface involvement is powerful, and ultrasound has a high diagnostic value in predicting the integrity of articular surface in patients. The obtained results confirmed that both ultrasound and arthrography are effective imaging modalities for predicting the integrity of the articular surface, but the former method is less invasive and does not lead to radiation exposure.

In the present study, even when the displacement of the fracture was ≥ 2 mm, 64.1% of the minimally displaced LHCFs had intact articular surfaces. Consistent with the findings of previous studies [2, 5, 7, 17], fractures displaced by < 4 mm on radiographs were more likely to have intact articular surfaces. However, no fractures with ≥ 4 mm of displacement were assessed by arthrography in Weiss’s study. Song et al. [15] found that all patients with incongruent articular surfaces had fractures displaced by > 2 mm, as measured by radiography. However, in Song’s study, the integrity of the cartilage hinge was mainly determined on the basis of the internal oblique radiograph. Although there was a statistically significant correlation between the arthrography assessments and fracture displacement, this correlation was not found in the ultrasound assessments. It is difficult to assess the relationship between the displacement of the fracture and the integrity of the cartilage hinge. In particular, there were only three patients with > 4 mm of fracture displacement in this study. We did not find a relationship between fractures with > 4 mm of displacement and the integrity of the articular surface in our study. However, this 4-mm cutoff value was not a clinical criterion prospectively used for the assessment of the incongruity of the articular surface. In addition, compared with the assessment of the displacement of the fracture, the routine use of ultrasound was more effective in evaluating the cartilage hinge status before the initial treatment of these fractures.

Three patients were predicted to have different statuses of articular surface integrity according to the ultrasound and arthrography assessments. Ultrasound can be used to observe the hypoechoic layer of the hyaline articular cartilage in the distal humeral epiphysis. The fracture line is directly identified by the hyperechoic gap and the disrupted hypoechoic layer on the anterior articular surface [10]. Arthrography is a reference standard for predicting the integrity of the articular cartilage surface [2, 18]. However, arthrography indirectly detects the integrity of the articular surface of the distal humerus through contrast medium tracking. It is difficult to assess complex three-dimensional articular cartilage fractures by arthrography. In addition, arthrography leads to radiation exposure, which requires sedation or anesthesia, and false-negative results have been reported [19]. Pennock et al. [17] suggested that arthrography findings are unclear and cannot be used to confirm the congruency of the articular surface. Although this study did not confirm these differences, on the basis of our data, we believe that ultrasound can provide more accurate information to determine the integrity of the articular cartilage.

In the present study, CRPP was performed in all patients, and no major complications occurred. As previously reported in the literature, whether minimally displaced LHCFs should be treated with ORIF or CRPP is controversial. Displaced LHCFs with displacements > 1 mm were treated with ORIF to avoid re-displacement and non-union, enabling direct visualization of the articular surface to confirm anatomical reduction [20]. Because the articular surface was intact in most cases displaced by < 4 mm, as confirmed by arthrography, these fractures were recently treated safely using CRPP, and no major complications were reported [2, 17]. Song et al. [15, 21] expanded the indications of CRPP to all fractures with incongruent articular surfaces or fracture displacements > 2 mm, with a closed reduction success rate of 73% (46/63). In particular, in patient number 24 (Fig. 1), the displacement was measured to be 5 mm on the internal oblique radiographs. The articular cartilage was confirmed to be intact by ultrasound. Elbow fractures can easily be reduced without surgery by inducing overstretching and a valgus angle of the elbow joint because the elbow has intact cartilage hinges. Therefore, we recommend that CRPP is included in the treatment of LHCFs with minimal displacement, especially in patients with an intact articular surface.

Our study has some limitations. First, although this is the largest study on this topic published to date, the sample size is still small. Our results clearly show that ultrasound and arthrography yielded consistent results in predicting the integrity of cartilage hinges of children with min-displaced LHCFs. We believe that our results can be generalizable to cases treated by other clinicians with focused ultrasound. Second, an inherent limitation of this study is that arthrography was related with the diagnostic criteria of ultrasound in predicting the integrity of the cartilage hinge and the stability of fractures. In fact, whether arthrography itself meets the diagnostic criteria has not yet been reported. However, we performed arthrography for all fractures according to the standard procedure. Recently, to research this issue further, we began using preoperative MRI and ultrasound to better assess the integrity of the articular surface of fractures.

## Conclusion

This report confirmed that ultrasound plays an important role in the diagnosis and treatment of fractures in children. A significant correlation was found between ultrasound and arthrography findings in assessing the integrity of the articular cartilage in the distal humerus. In addition, compared with arthrography, ultrasound is noninvasive, simple, and effective. Ultrasound can be used as a complementary tool with arthrography for preoperative assessment of the integrity of the cartilage hinge in children with minimally displaced LHCFs.

## Data Availability

The datasets used and/or analyzed during the current study are available from the corresponding author on reasonable request.

## Abbreviations

LHCFs: Lateral humeral condyle fractures
ORIF: Open reduction and internal fixation
CRPP: Closed reduction and percutaneous pinning
MRI: Magnetic resonance imaging
